# Identification and functional analysis of proteins in response to light intensity, temperature and water potential in *Brassica rapa* hypocotyl

**DOI:** 10.1111/ppl.12865

**Published:** 2019-01-10

**Authors:** Hongfei Wang, Qingmao Shang

**Affiliations:** ^1^ Key Laboratory of Biology and Genetic Improvement of Horticultural Crops, Ministry of Agriculture Institute of Vegetables and Flowers, Chinese Academy of Agricultural Sciences Beijing China

## Abstract

Hypocotyl elongation is an early event in plant growth and development and is sensitive to fluctuations in light, temperature, water potential and nutrients. Most research on hypocotyl elongation has focused on the regulatory mechanism of a single environment factor. However, information about combined effects of multi‐environment factors remains unavailable, and overlapping sites of the environmental factors signaling pathways in the regulation of hypocotyl elongation remain unclear. To identify how cross‐talks among light intensity, temperature and water potential regulate hypocotyl elongation in *Brassica rapa* L. ssp. *chinesis*, a comprehensive isobaric tag for relative and absolute quantitation‐based proteomic approach was adopted. In total, 7259 proteins were quantified, and 378 differentially expressed proteins (DEPs) were responsive to all three environmental factors. The DEPs were involved in a variety of biochemical processes, including signal transduction, cytoskeletal organization, carbohydrate metabolism, cell wall organization, protein modification and transport. The DEPs did not function in isolation, but acted in a large and complex interaction network to affect hypocotyl elongation. Among the DEPs, phyB was outstanding for its significant fold change in quantity and complex interaction networks with other proteins. In addition, changes of sensitivity to environmental factors in *phyB‐9* suggested a key role in the regulation of hypocotyl elongation. Overall, the data presented in this study show a profile of proteins interaction network in response to light intensity, temperature and water potential and provides molecular basis of hypocotyl elongation in *B. rapa*.

**Abbreviations**
2‐DE: 2‐dimentional electrophoresis
β‐GLU: β‐glucosidase
ACN: acetonitrile
CCOMT: caffeoyl‐O‐methyltransferase
COP1: constitutive photomorphogenic 1
DEPs: differentially expressed proteins
GO: gene ontology
HY5: elongated hypocotyl 5
iTRAQ: isobaric tags for relative and absolute quantitation
LC/LC–MS/MS: liquid chromatography tandem mass spectrometry
PCAP1: plasma‐membrane‐associated cation‐binding protein 1
phyB: phytochrome B
PIF4: photochrome interacting factor 4
RPLC: reversed‐phase liquid chromatography

## Introduction

Living in a complicated and changeable environment, higher plants have developed a sophisticated system to collect and integrate information provided by light, temperature, water potential, nutrients, and so on (Sun et al. 2012, Toledo‐Ortiz et al. 2014). The combined signals are translated into efficient acclimation to optimize seedlings architecture (Lorenzo et al. 2016). As a pivotal morphologic trait, hypocotyl growth facilitates the soil penetration of germinating seeds, assists the seedlings in competing for better light harvest and helps seedlings reducing the risk of heat and water stress on soil surface (Wu et al. 2005, Lorrain et al. 2008, Franklin et al. 2011, Procko et al. 2014). However, leggy seedlings with over‐elongated hypocotyls exhibit reduced defenses against pathogens and herbivores (Procko et al. 2014). To protect seedlings from over‐elongating and improve our understanding of the hypocotyl elongation mechanism, extensive researches have been conducted and some key signaling factors have been revealed, such as PHYTOCHROME B (PhyB), CONSTITUTIVE PHOTOMORPHOGENIC 1 (COP1), ELONGATED HYPOCOTYL 5 (HY5), PHOTOCHROME INTERACTING FACTOR 4 (PIF4) and so on (Franklin et al. 2011, Sun et al. 2012, Lu et al. 2015, Ma et al. 2016).

PhyB, acting as both a light and temperature receptor, plays a central role in seed germination, seedling de‐etiolation and resistance to pathogens (Reed et al. 1993, Li et al. 2015). The *phyB* mutant has been reported in many species, such as *Arabidopsis thaliana*, tomato (*Solanum lycopersicum* L.), pea (*Pisum sativum* L.), rice (*Oryza sativa* L.), and all these mutants displayed symptoms of shade‐avoidance syndrome, including closed cotyledons, increased hypocotyl and petiole length, reduced root and leaf growth (Reed et al. 1993, Weller et al. 1997, Lazarova et al. 1998, Takano and Shinomura 2005). Evidence emerges that low light, shade and high temperature promote hypocotyl elongation by repressing protein abundance of PhyB, which controls plant hormones biosynthesis and signaling transduction (Stavang et al. 2009, Sun et al. 2012, Delker et al. 2014, Xu et al. 2018, Yang et al. 2018).

Proteomic approaches have provided insights into the changes in plant organizations in response to the stimuli at the protein level and have been widely used to study the hypocotyl elongation mechanism (Khan et al. 2015, Luo et al. 2015). Feiz et al. (2006) and Irshad et al. (2008) analyzed the cell wall proteome as the hypocotyl elongated in *A. thaliana* using traditional 2‐dimensional electrophoresis (2‐DE), and differentially expressed proteins (DEPs) involved in cell wall modification and lipid metabolism were identified. However, the number of DEPs was strictly limited by the sensitivity. To overcome the shortcomings of 2‐DE, technology using isobaric tags for relative and absolute quantitation (iTRAQ) has been extensively used to detect protein concentrations in complex systems (Zhai et al. 2017). Therefore, we analyzed the DEPs in the hypocotyl of *Brassica rapa* L. ssp. *chinesis* under the combined effects of light intensity, temperature and water potential using iTRAQ.

*B. rapa* is an economically important vegetable crops, and hypocotyl elongation is sensitive to fluctuations in environment factors (Procko et al. 2014). Additionally, the close relationship of *B. rapa* with the model plant *A. thaliana* and detailed genomic information make it ideal for proteomic studies. Combined with iTRAQ technology, we explored the DEPs and pathways in response to light intensity, temperature and water potential in hypocotyls. As a result, 7259 proteins were successfully identified, and 378 showed significant difference in quantity and were simultaneously regulated by the three environmental factors, including PhyB, which acted as a temperature and photoreceptor. Functional analysis indicated that the 378 DEPs played a role in signal transduction, carbohydrate metabolism, cell wall organization, etc. The signal transduction involving PhyB played a key role in regulating hypocotyl extension, and hypocotyl phenotypes in *phyB‐9* strengthened the result. Our data not only provided a new insight into the complex function of PhyB but also laid a molecular basis for hypocotyl elongation under the combined effects of light, temperature and water potential.

## Materials and methods

### Plant materials and growth conditions

**Figure 1:**
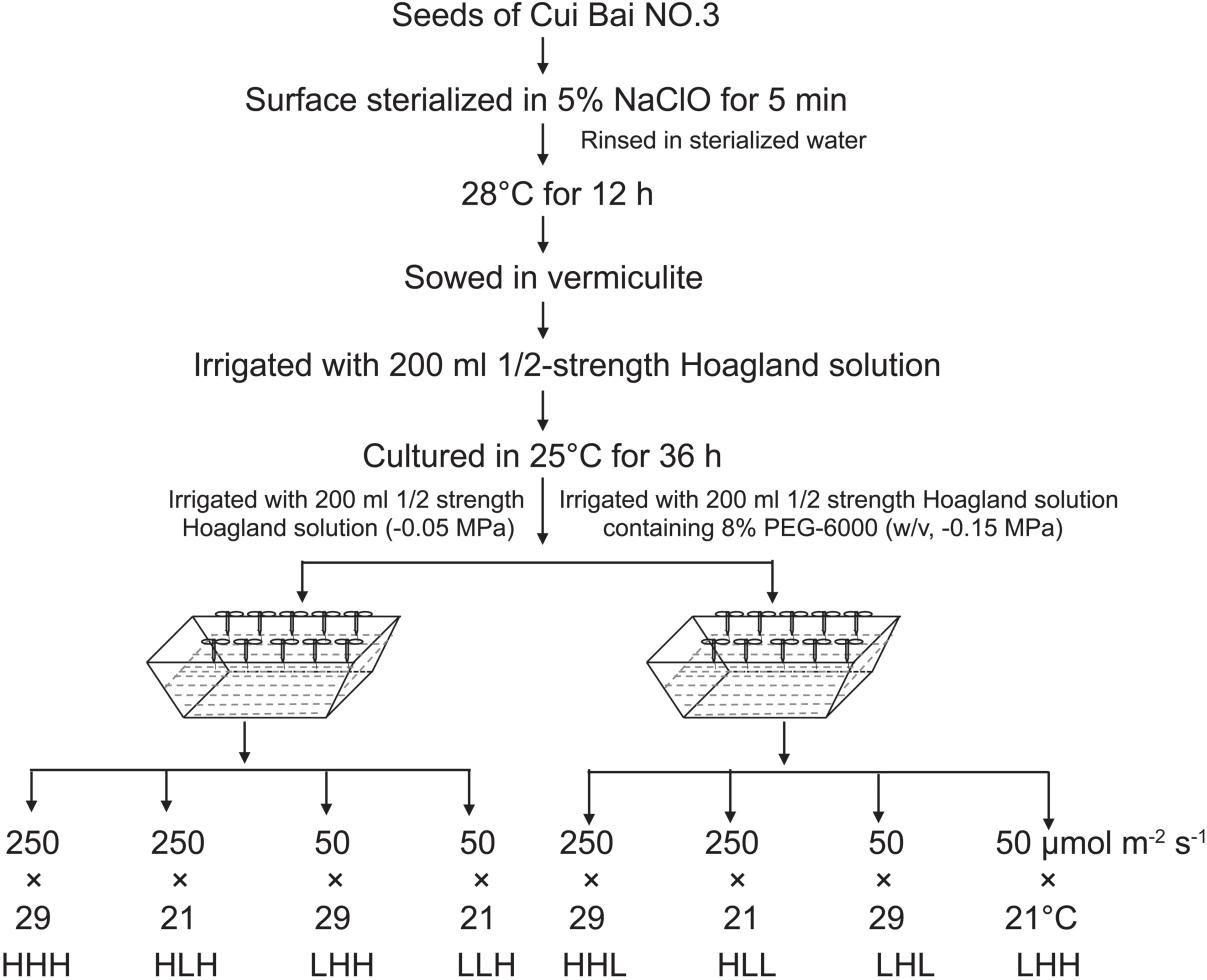
Workflow of seedling treatments.

Seeds of *B. rapa* (cv. CuiBai No. 3) were surface‐sterilized in 5% sodium hypochlorite and washed using sterile water. Germinated seeds were sown in vermiculite watered with 150 ml 1/2‐strength Hoagland solution, and cultured at 25°C in the dark for 36 h. The seedlings were used for single and complex environmental factor experiments. For the light intensity assay, the seedlings were watered with 200 ml 1/2‐strength Hoagland solution or solution containing 8% polyethylene glycol 6000 (w/v; PEG‐6000; Sinopharm, Beijing, China) and transferred to chambers with different irradiances (50, 100, 150, 200 and 250 µmol m^−2^ s^−1^; 16‐h photoperiod). In the temperature treatments, the seedlings were watered with two kinds of solutions as described above and grown in differentially warmed chambers (17, 21, 25, 29 and 33°C), and the temperatures were kept constant day and night. The seedlings used for water potential trial were watered with 200 ml 1/2‐strength Hoagland solution containing gradient PEG‐6000 (0, 4, 8, 12, and 16%). The water potentials of gradient solutions were assayed using Psypro water potential system (Wescor, Logan, UT), and the osmotic potential was calculated as described by Michel and Kaufmann (1973) (Table S1, Supporting information). For the analysis of the combined effects of light intensity, temperature and water potential on hypocotyl elongation, seedlings watered with 200 ml 1/2‐strength Hoagland solution or solution containing 8% PEG‐6000 were transferred to the chambers with different light intensities and temperatures (Fig. 1) and HHL, HHH, HLL, HLH, LHL, LHH, LLL, LLH in Fig. 1 represented ‘high light, high temperature, and low water potential’, ‘high light, high temperature, and high water potential’, ‘high light, low temperature, and low water potential’, ‘high light, low temperature, and high water potential’, ‘low light, high temperature, and low water potential’, ‘low light, high temperature, and high water potential’, ‘low light, low temperature, and low water potential’, ‘low light, low temperature, and high water potential’, respectively. After 40 h, the hypocotyls of the seedlings were harvested for proteomic assay in three biological replicates.

The seeds of *phyB‐9* mutant (Reed et al. 1993) and wild‐type Columbia (Col‐0) in *A. thaliana* were surface‐sterilized and sown on Murashige and Skoog (MS) plates containing 4.43‐g l^−1^ MS salts (Caisson Laboratories, Smithfield, UT), 1.2‐g l^−1^ 2‐N‐morpholino‐ethanesulfonic acid (Biodee, Beijing, China), and 0.8% w/v agar, pH 5.7. The plates were incubated at 4°C for 2 days and cultured at 25°C for 2 days. Half of the seedlings were transferred to the new MS plates or MS plates infused with PEG‐6000 solutions (Cm et al. 2000, Verslues et al. 2006). The transplanted seedlings were transferred to controlled chambers, as shown in Fig. 1, for 5 days.

### Phenotype measurement

The hypocotyl length in *B. rapa* was measured using a millimeter scale (accuracy ± 0.5 mm), and 60 plants were scored in three independent replicates. The hypocotyl length of *A. thaliana* seedlings was assayed in three independent replicates (60 plants) using the imagej software (http://rsbweb.nih.gov/ij/) (NIH, Bethesda, MD). Relative hypocotyl length induced by low light was calculated as follows:

Relative hypocotyl length = LHL/HHL; LHH/HHH; LLL/HLL; LLH/HLH.

Relative hypocotyl length induced by high temperature = HHL/HLL; HHH/HLH; LHL/LLL; LHH/LLH. Relative hypocotyl length induced by high water potential = HHH/HHL; HLH/HLL; LHH/LHL; LLH/LLL.

### Protein extraction

Hypocotyl tissue from *B. rapa* seedlings grown under the combined effects of different light intensity levels, temperatures and water potentials for 40 h was harvested and stored in −80°C. Hypocotyl tissues from 60 plants were used in each sample, as well as three replicates in per sample. The total proteins were extracted from the hypocotyls by trichloroacetic acid‐acetone as previously described with some modification (Hao et al. 2012). The mixtures were purified using acetone, and the purified proteins were added to lysis buffer (7‐M urea, 2‐M thiourea, 4% 3‐[3‐(cholylamide propyl) dimethylamino] propionic sulfonic acid (CHAPS), and 40‐mM Tris–HCl, pH 8.5). The concentration of the extracted protein was determined using the Bradford method (Bio‐Rad, Hercules, CA; Bradford 1976), and the quality was analyzed using sodium dodecyl sulfate polyacrylamide gel electropheresis (SDS‐PAGE). The protein samples were stored at −80°C for further analysis.

### Protein digestion and iTRAQ labeling

The extracted proteins (100 µg) of the samples were reduced with 20 mM dithiothreitol (DTT) at 37°C for 60 min, and alkylated with 40 mM iodoacetamide (IAA) for 40 min in darkness at 25°C. The alkylated protein sample was digested at a trypsin: protein mass ratio of 1:25 overnight at 37°C. Digested peptides were labeled with iTRAQ reagents using the manufacturer's instructions (Applied Biosytems, Foster City, MA). The peptides extracted from the *B. rapa* hypocotyls grown in HHL, HHH, HLL, HLH, LHL, LHH, LLL, and LLH were labeled with iTRAQ tags of 113, 114, 115, 116, 117, 118, 119, and 121, respectively. The three biological replicates of each sample were separately labeled and used for LC/MS–MS and further independent analysis. The labeled samples were pooled and dried in a rotary vacuum concentrator (Christ RVC 2–25; Christ, Osterode, Germany).

### Liquid chromatography tandem mass spectrometry analysis

The additional analysis used liquid chromatography tandem mass spectrometry (LC/LC–MS/MS) as previously described with some modifications (Nogueira et al. 2012). The labeled peptides were diluted with loading buffer (5 mM ammonium formate), including 2% acetonitrile (ACN, pH = 10) and separated using high‐pH reversed‐phase liquid chromatography (RPLC, Acquity Ultra Performance LC; Waters Corporation, Milford, MA). The peptides were eluted at a flow rate of 400 µl min^−1^ using a gradient elution of 0 to 25% B (0 to 53 min), 25 to 80% B (53 to 65 min) on a high‐pH RPLC column (C18, 3.5 µm, 150 × 2.1 mm; Waters, Milford, MA). The 40 collected fractions were pooled into 10 fractions and vacuum‐dried.

The fractions collected were separated and analyzed in more detail using LC/MS–MS with a Nano Aquity Ultra Performance Liquid Chromatography (UPLC) system (Waters, Milford, MA) equipped with a Q Exactive hybrid quadrupole‐Orbitrap mass spectrometer (Thermo Fisher Scientific, Waltham, MA). The peptides of each sample (8 µl), dissolved in solvent A (2% ACN containing 0.1% formic acid) were loaded on a C18 column (75 µm × 25 cm; Thermo Fisher Scientific, Waltham, MA) at a flow rate of 300 nl min^−1^. The peptides on an analytical column were eluted by gradient solvent B: from 5 to 48% B over 88 min, followed by 100% B for the next 1 min, and then held for 7 min. The column was equilibrated for 25 min in the initial condition. The eluents were transferred to the Q Exactive mass spectrometer operated in the data‐dependent mode to switch automatically between MS and MS/MS acquisition with a full scan MS spectra (350–1300 m/z), acquired with a resolution of 70 000, and an MS/MS scan was followed with a resolution of 17 500.

### Protein identification, quantification and annotation

The raw data from the LC/LC–MS/MS were analyzed using Proteome Discoverer TM 2.1 (PD + sequest) (Thermo Fisher Scientific, Waltham, MA). The processed data were used to search for proteins using the Mascot search engine (Matrix Science, London, UK) against the *B. rapa* (http://brassicadb.org/brad/index.php) and *Brassica oleracea* database (http://ocri-genomics.org/bolbase/index.html). Proteins were identified as follows: sample type = iTRAQ 8‐plex (peptide‐labeled), Cys; alkylation = carbamidomethyl; digestion = trypsin; instrument = ProteomeDiscverer version (2.1); database = Brassica_rapa.20100830.pep.

The proteins, qualified with at least one unique peptide and a false discovery rate < 0.05, were used for quantification analysis. In this research, we set the threshold as *P* < 0.05 and fold change >1.2 to classify the DEPs into the significance level. A significance evaluation of the comparison between the two groups in each repetition was conducted using the Student's *t*‐test. The DEPs were annotated further according to Gene Ontology (GO) and Kyoto Encyclopedia of Genes and Genomes (KEGG) analysis.

### RNA extraction and quantitative real‐time polymerase chain reaction

Hypocotyls for the proteomics analysis were used for total RNA extraction using the TRIzol reagent (Invitrogen, Carlsbad, CA) according to the manufacturer's instructions. A total of 1.5 µg RNA was reverse transcribed into cDNA using a Reverse Transcription System (Promega, Madison, WI). Representative DEPs from key functional groups were selected to analyze their expression abundance at the transcript level. Gene‐specific primers (Table S2) were designed using Primer Premier 5.0 Software (Premier Biosoft, Palo Alto, CA). The quantitative reverse transcription polymerase chain reaction (qRT PCR) was performed using a LightCycler 96 real‐time PCR system (Roche, Basel, Switzerland), and the relative expression level was calculated by the 2^‐ΔΔCt^ method (Schmittgen and Livak 2008) using *GAPDH* as the reference gene (Qi et al. 2010, Procko et al. 2014).

## Results

### Combined effects of light intensity, temperature and water potential on hypocotyl elongation

**Figure 2:**
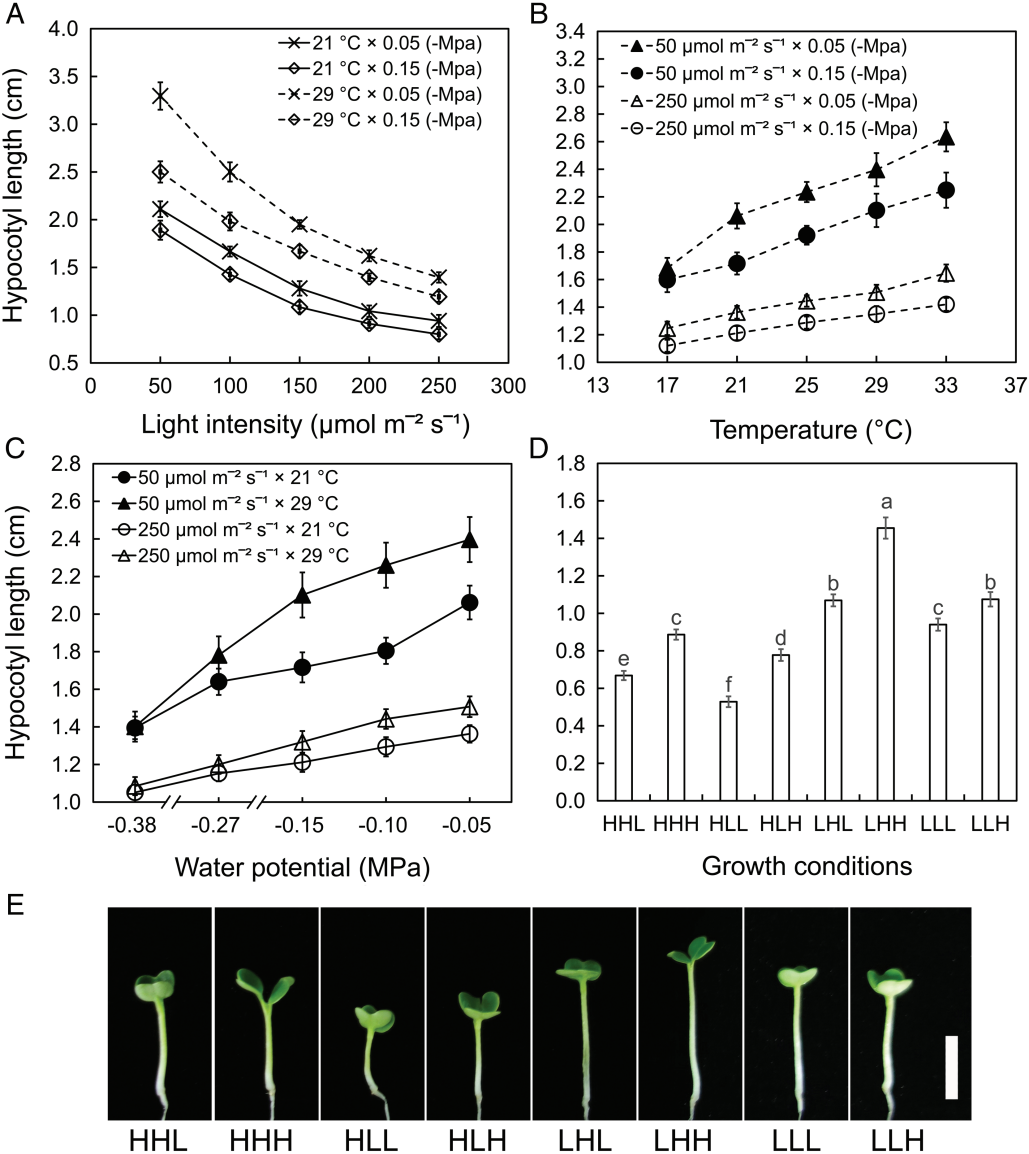
The influence of light intensity, temperature and water potential on hypocotyl elongation in *B. rapa*. (A) High light represses hypocotyl elongation. (B) Hypocotyl elongates incrementally as the temperature increases. (C) Hypocotyl elongates incrementally as the water potential increases. (D) Hypocotyl length of *B. rapa* seedlings under the combined effects of light intensity, temperature and water potential for 40 h. The data in A‐D represent the means of three replicates ± sd. Small letters in D represent significant differences among the seedlings under different conditions (*P* < 0.05). (E) Photograph of *B. rapa* seedlings under the combined effects of light intensity, temperature and water potential for 40 h. Scale bar represents 1 cm.

The hypocotyls of *B. rapa* elongated incrementally and significantly as the light intensity decreased in intervals of 50 µmol m^−2^ s^−1^, especially when the intensity was lower than 150 µmol m^−2^ s^−1^ (Fig. 2A). The effects of the temperature treatments on hypocotyl elongation gained significance when the higher temperatures, 21, 25, 29 and 33°C, were compared with the lower temperature 17°C (Fig. 2B). Hypocotyl elongation induced by high water potential was significant across the entire range (−0.38, −0.27, −0.15, −0.10, −0.05 MPa), and the increases in hypocotyl length were larger when the water potential improved from −0.38 to −015 MPa than when the water potential ranged from −0.15 to −0.05 MPa (Fig. 2C). The influence of light intensity on hypocotyl elongation was highly dependent on temperature and water potential, and the maximum increase in hypocotyl length induced by the low light (50 µmol m^−2^ s^−1^) occurred at 29°C and −0.05 MPa. The ability of temperature to promote hypocotyl elongation was tightly dependent on the light intensity and water potential. The maximum effect was detected at 50 µmol m^−2^ s^−1^ and −0.05 MPa. Elevated water potential promoted hypocotyl elongation across a wide range of light intensities and temperatures, and the greatest response was observed as the light intensity reduced to 50 µmol m^−2^ s^−1^ and the temperature increased to 29°C. The results indicated that light intensity, temperature and water potential regulated hypocotyl elongation in *B. rapa* not only in isolation but also in synergy.

Based on the results that single environmental factors regulate hypocotyl elongation, different light intensities (50/250 µmol m^−2^ s^−1^), temperatures (21/29°C) and water potentials (−0.05/−0.15 MPa) were selected to analyze the combined effects of the environmental factors. The difference in hypocotyl length among the seedlings under the combined effects of light intensity, temperature and water potential was significant (Fig. 2D, E). Interaction analysis of the three environmental factors was conducted based on the hypocotyl phenotypes. Significant interactions including two factors (‘light intensity and temperature’ and ‘light intensity and water potential’) and the interaction containing three factors (light intensity, temperature and water potential) were revealed (Table S3). To explore the underlying mechanism of environmental factors on hypocotyl elongation, the high‐throughput technology of iTRAQ was used to analyze the proteomic profiles in response to light intensity, temperature and water potential.

### Protein characteristics of the total proteins identified in hypocotyls

To get insight into molecular mechanism of hypocotyl elongation in response to light intensity, temperature and water potential, the proteome expression profiles were analyzed. Approximately, 371 445 spectra were obtained in which 113 955 spectra were matched to 42 380 known peptides (Table S4). In total, 7718 proteins were identified according to the peptides, in which 7259 proteins that were detected in two or three biological replications were used for further analysis (Table S5). The molecular weights of the proteins identified using iTRAQ ranged from 5.63 (Bra030248, signaling response 1) to 611.49 kDa (Bra034020; MIDASIN 1), among which the highest area of distribution was 1 to 100 kDa in size (Fig. S1). The results indicated that the iTRAQ was high‐throughput technology with a high degree of sensitivity, and it can obtain more comprehensive information to analyze the proteins in the hypocotyls of *B. rapa*.

**Figure 3:**
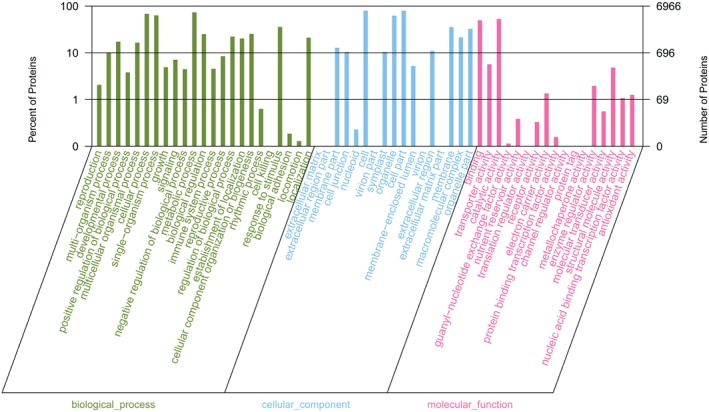
Functional categories of the total proteins identified in hypocotyl under the combined effects of light intensity, temperature and water potential using GO analysis.

**Figure 4:**
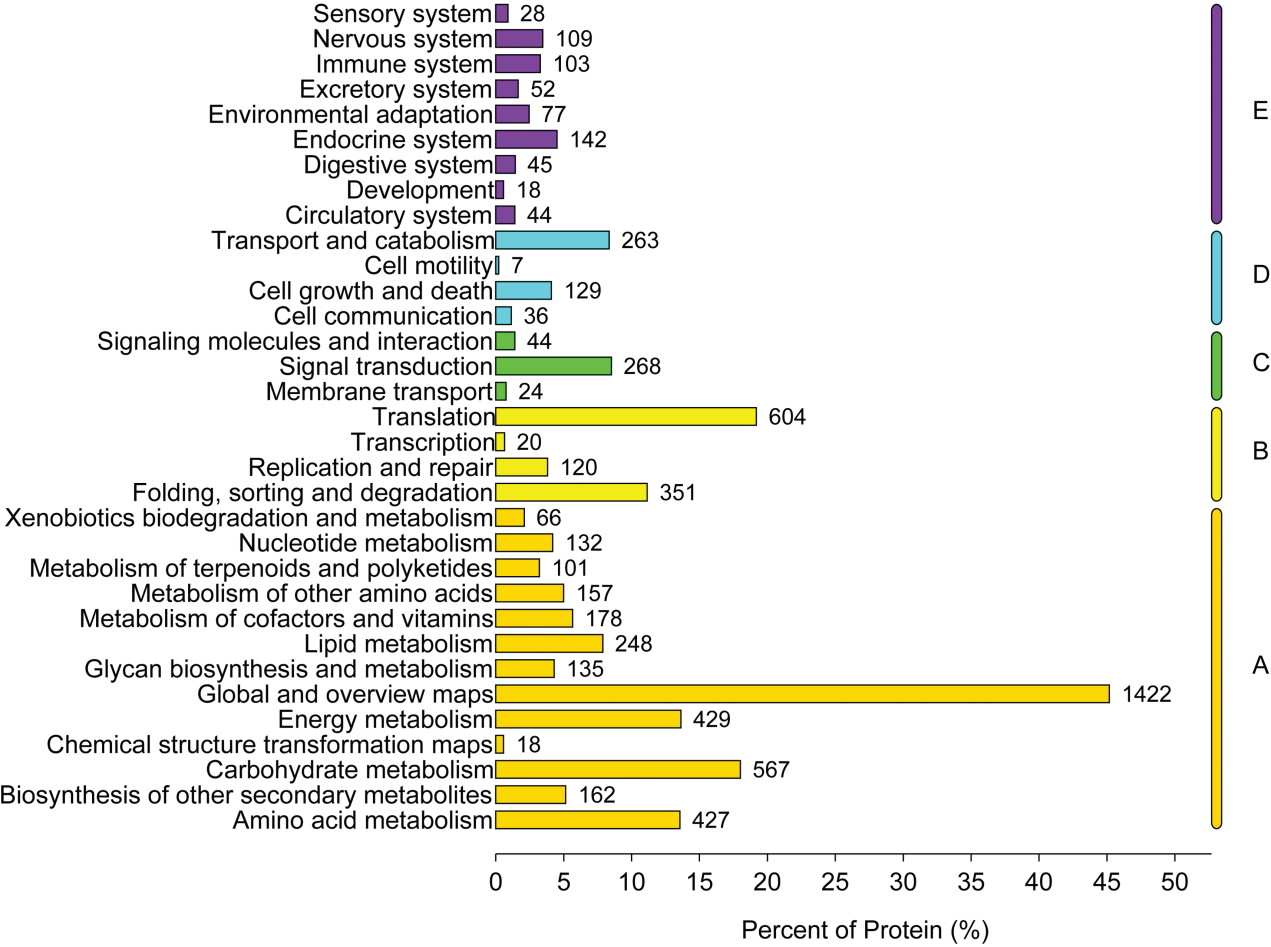
KEGG classifications of the total proteins identified in the hypocotyl under the combined effects of light intensity, temperature and water potential. The digit on the right of the bar indicates the protein number in each category. The letters on the right of the figure represent the KEGG pathways as follows: (A) metabolism; (B) genetic information processing, (C) environmental information processing, (D) cellular processes and (E) organismal systems.

All the proteins identified in the hypocotyls were quantified and analyzed using GO (Fig. 3) and the KEGG classification (Fig. 4), respectively. These proteins functioned in signaling, organelle, binding and transporting activity, which were categorized into biological process, cellular component and molecular function. According to the KEGG analysis, the proteins quantified were primarily involved in carbohydrate metabolism (568 proteins), translation (586 proteins), signal transduction (266 proteins) and transport and catabolism (252 proteins), indicating that complicated metabolism was highly active in the hypocotyls. The large number of identified proteins and detailed functional information enabled us to investigate the changes that occurred in the hypocotyls under the regulation of light intensity, temperature and water potential.

### Analysis of DEPs

**Figure 5:**
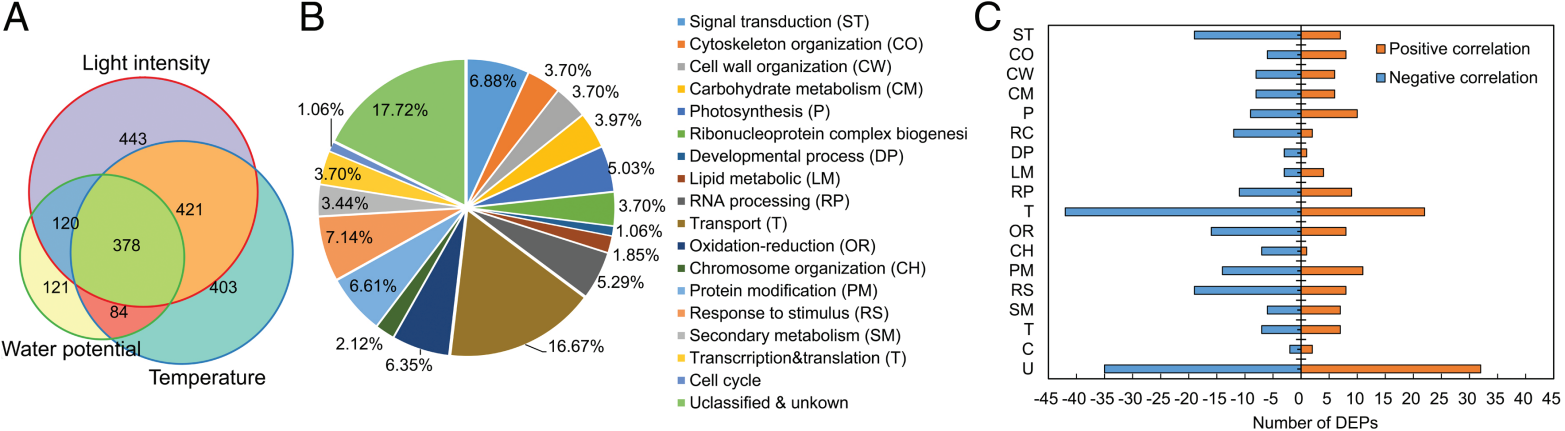
DEPs identification and functional analysis. (A) Identification of the DEPs responsive to light intensity, temperature and water potential. (B) Functional analysis of the 378 DEPs according to the GO and KEGG database. (C) The number of DEPs positively and negatively related to hypocotyl length in each category. The abbreviations of functional categories were indicated in picture (B). The DEPs represent differentially expressed proteins.

To identify responsive proteins and pathways during hypocotyl elongation regulated by light intensity, temperature and water potential, DEPs were analyzed using the standard fold change ≥1.2 and *P* ≤ 0.05. A total of 1362 DEPs induced by light intensity were identified based on binary comparison (LHL vs HHL and LHH vs HHH and LLL vs HLL and LLH vs HLH). A total of 1286 proteins were selected as DEPs in response to temperature (HHH vs HLH and HHL vs HLL and LHH vs LLH and LHL vs LLL). In contrast, only 703 proteins were defined as DEPs influenced by water potential (HHH vs HHL and HLH vs HLL and LHH vs LHL and LLH vs LLL). To detect the DEPs in response to light intensity, temperature, and water potential, Venny 2.1.0 (http://bioinfogp.cnb.csic.es/tools/venny/index.html; CNB, Porto, Spanish) was used (Fig. 5A). A total of 799 DEPs both responded to light intensity and temperature; 499 DEPs were under the coregulation of light intensity and water potential and the expression levels of 462 DEPs were deeply influenced by the temperature and water potential. In addition, 378 DEPs responded to light intensity (27.75%), temperature (29.39%) and water potential (53.39%; Table S6).

Based on functional annotation (GO) and metabolic pathway (KEGG) analysis, the 378 DEPs were categorized into 16 major types, including signal transduction (6.88%), cytoskeleton organization (3.70%), cell wall organization (3.70%), carbohydrate metabolic (3.70%), responding to stimulus (7.14%) and transport (16.93%) (Fig. 5B). The number of DEPs negative to hypocotyl elongation (227 DEPs) was larger than those positive to hypocotyl length (151 DEPs; Fig. 5C). In addition, the negatively related proteins were primarily involved in signal transduction, carbohydrate metabolism, cell wall organization, oxidation–reduction metabolism and transport. The positively related proteins tended to be enriched in the pathways of cytoskeleton organization, water transport, secondary metabolism and nucleotide metabolism, which coincide with the symptoms of seedlings growth (Fig. 2E). We concluded that light intensity, temperature and water potential shared some signaling factors and pathways to regulate hypocotyl elongation, and these signaling proteins in response to the three environmental factors were involved in nearly every aspect of plant growth and development.

Among the negatively related proteins, PhyB that contributed to both light and temperature signal transduction (Casal and Questa 2018) was outstanding for its significant fold change in quantity and complex interaction network with other DEPs. In our research, PhyB functioned to regulate the hypocotyl elongation of *B. rapa* under the influence of light intensity, temperature and water potential. In addition, it was in higher abundance in high light (1.24‐fold), low temperature (1.32‐fold) and high water potential (1.30‐fold). This result indicated that PhyB could function as convergence of light, temperature and water potential signals in the regulation hypocotyl growth. In addition, the fold change in the quantity of PhyB suggested that the hypocotyl, considered to act based on endogenous signals from the cotyledons, could sense environment fluctuations directly and adjust growth itself.

### DEPs interaction network

**Figure 6:**
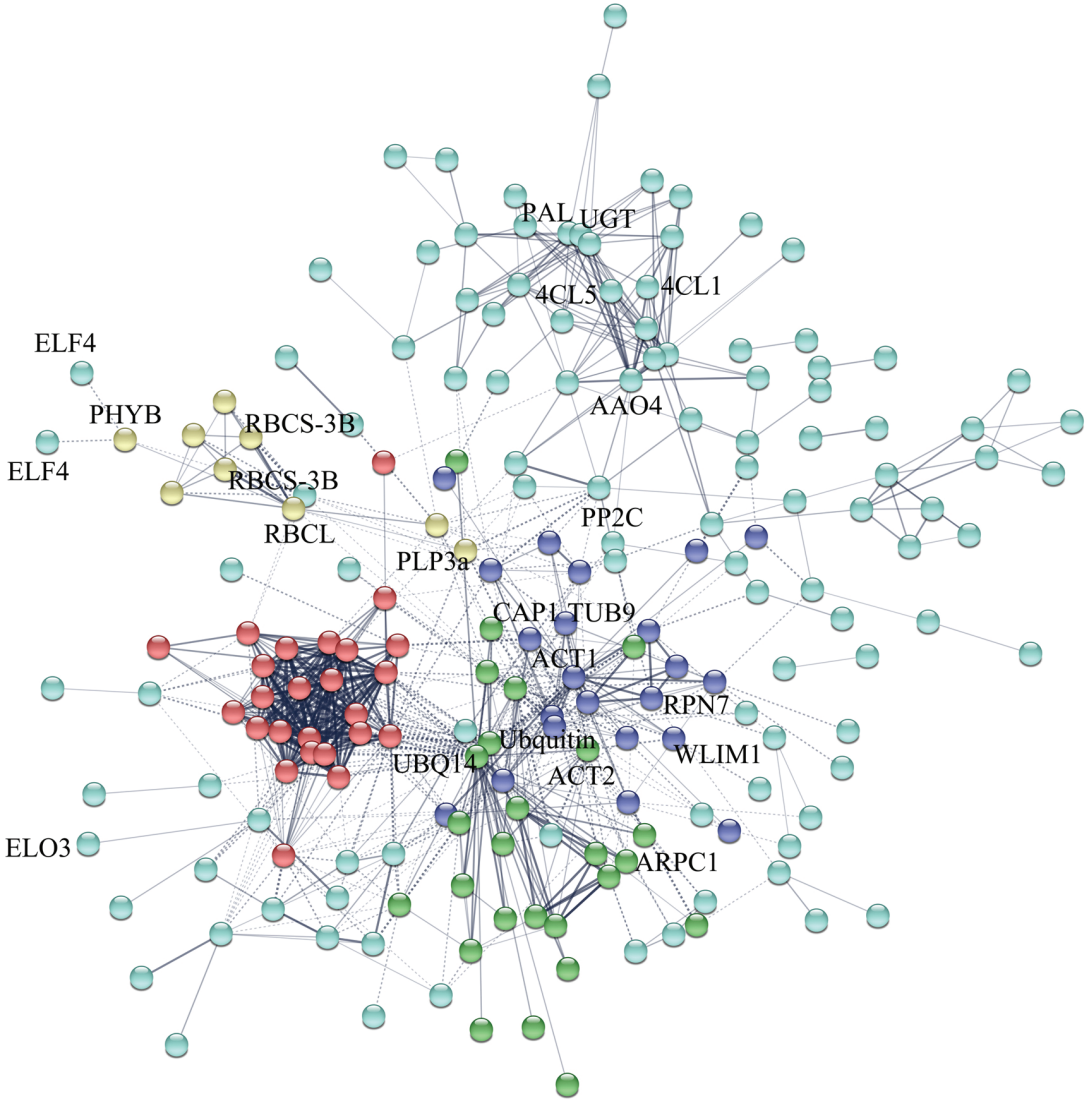
Protein–protein interaction networks of 378 DEPs in the *B. rapa* hypocotyl. Nodes of different colors represent proteins. Edges represent protein–protein association. The coarseness of the lines represents interaction strength. Solid lines in the picture indicate protein interaction in intragroup. Dotted lines represent protein interaction between groups.

The protein–protein interaction network was constructed to further analyze the molecular mechanism of hypocotyl elongation in *B. rapa*, and 187 of 378 DEPs demonstrated a direct linkage with each other. Based on the strength of the protein associations, these DEPs were categorized into five classifications. A total of 109 proteins (107 belonging to the DEPs) that were identified in our research and primarily involved in the process of signal transduction, cell wall organization and carbohydrate metabolism in the network, offered strong interactions with the other members in the group, which were classified as class 1 (blue ball, Fig. 6). In group 2 (green ball, Fig. 6), 25 of the 26 proteins that functioned in cytoskeleton organization and protein modification, were differentially expressed. Twenty‐two proteins (21 were DEPs) were divided into group 3 (purple ball, Fig. 6) and had a close relationship with those of group 2. The members of group 4 (red ball, Fig. 6) that were primarily involved in ribonucleoprotein complex biogenesis exhibited strongest interaction with each other. In addition, nine DEPs involved in signal transduction and photosynthesis constituted the members of group 5 (yellow ball, Fig. 6). These results showed that the DEPs functioned together to regulate hypocotyl growth instead of acting in isolation.

### Transcriptional confirmation of DEPs using qRT‐PCR

**Figure 7:**
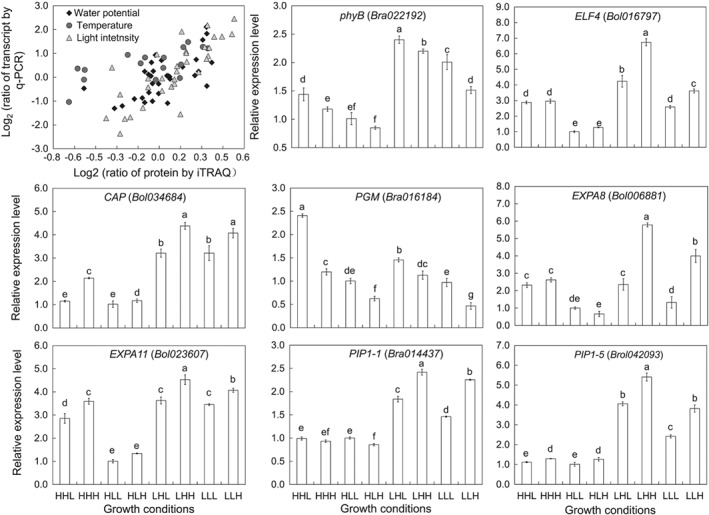
Transcriptional confirmations according to qRT‐PCR and relative expression level of maker genes involved in regulating hypocotyl elongation.

To further analyze the correspondence between the proteins and the mRNA expression levels of the DEPs, 59 proteins were selected to assay their transcriptional changes using qRT‐PCR based on the genome sequence of *B. rapa* (http://brassicadb.org/brad/index.php). The selected DEPs functioned in signal transduction, cell wall organization, cytoskeleton organization and photosynthesis. The comparison with the expression profiles of iTRAQ and qRT PCR indicated that most of the DEPs selected were confirmed to be reliable (Fig. 7). For example, the maximal fold changes of PIP1‐5 at the mRNA level induced by low light, high temperature and high water potential were 4.19, 2.31 and 2.48, respectively, and the corresponding fold changes at the protein level were 1.51, 1.43 and 1.40, respectively. However, the relative agreement of some proteins was poor, especially for the DEPs responding to temperature, such as HISTONE H2B (H2B.10), β‐GLUCOSIDASEE (β‐GLU), PHOSPHOGLUCOMUTASE and PLASMA MEMBRANE ASSOCIATED CATION BINDING PROTEIN 1 (PCAP1). In addition, post‐transcriptional and post‐translational regulation may contribute to the non‐concordance between the mRNA and protein levels as previously described (Chu et al. 2015).

### Functional analysis of key DEP

**Figure 8:**
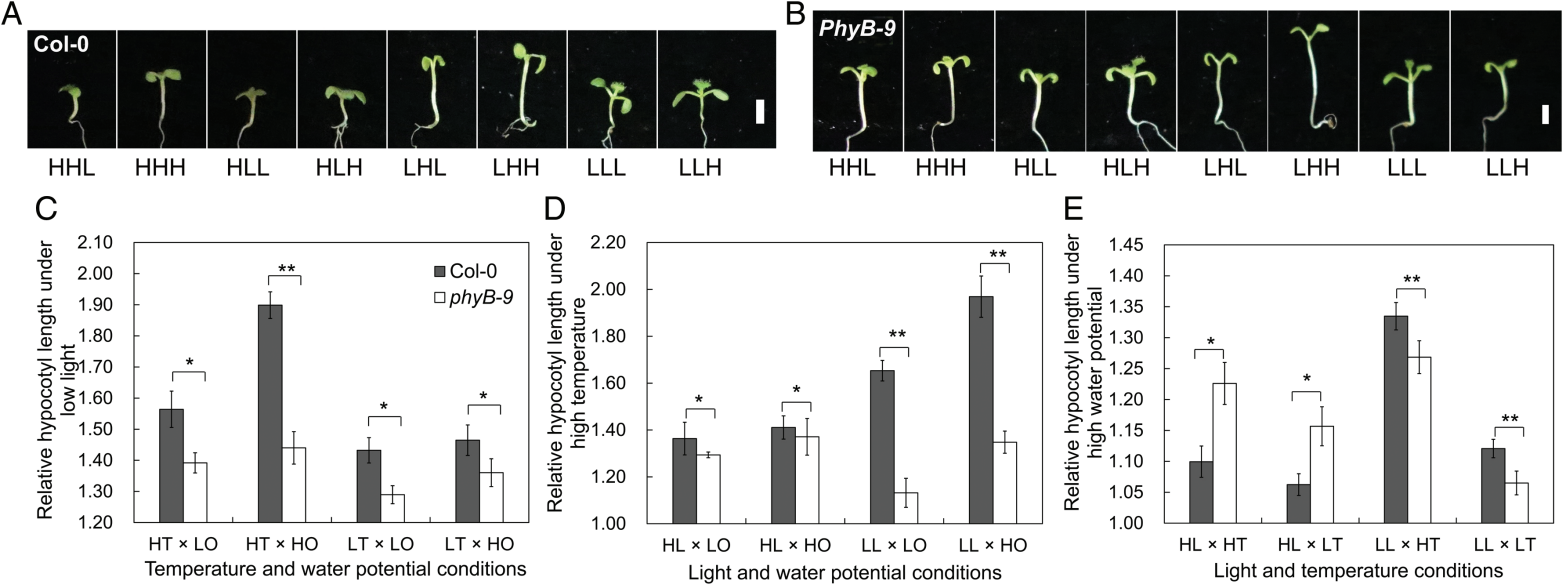
Hypocotyl phenotypes in Col‐0 and *phyB‐9* under the combined effects of light intensity, temperature and water potential. (A) Picture of the seedlings in Col‐0 under the combined effects of light intensity, temperature and water potential for 5 days. (B) Picture of the seedlings in *phyB‐9* under the synergy of light intensity, temperature and water potential for 5 days. (C) Hypocotyl elongation induced by low light in Col‐0 and *phyB‐9*. (D) Hypocotyl elongation responding to high temperature in Col‐0 and *phyB‐9*. (E) Hypocotyl of Col‐0 and *phyB‐9* elongated on different extent responding to high water potential. The data in C, D and E represented the means of three replicates ± sd. Asterisks represent significant difference between the mutant and wild type (*P < 0.05; **P < 0.01). In picture C‐E, HL and LL represent high light and low light respectively; HT and LT represent high temperature and low temperature; HO and LO represent high water potential and low water potential, respectively.

To evaluate the roles of the DEPs belonging to signal transduction in regulating hypocotyl elongation, functional analysis was conducted on the hypocotyl length of the wild‐type and *phyB‐9* mutant in response to light intensity, temperature and water potential in *A. thaliana*. The relative hypocotyl length of Col‐0 and the *phyB‐9* mutant was calculated, respectively. Hypocotyl elongation induced by low light, high temperature and high water potential both in Col‐0 and *phyB‐9* was detected (Fig. 8A, B). However, the increase in hypocotyl length induced by low light and high temperature was larger in Col‐0 than in *phyB‐9* (Fig. 8C, D). The influence of water potential on hypocotyl elongation was highly dependent on light intensity (Fig. 8E). The increase in hypocotyl length promoted by high water potential was larger in Col‐0 than in *phyB‐9* under high light, while it was larger in *phyB‐9* than in Col‐0 under low light. Even more, anova comparison of treatment × genotype was conducted (Table S7), and the result indicated genotype had significant influence of on hypocotyl elongation in response to light intensity, temperature and water potential. The interaction of genotype × light intensity, genotype × temperature and genotype × water potential also had significant influence on hypocotyl elongation.

To strengthen the function of PhyB in response to water potential, MS plates infused with gradient PEG‐6000 solutions were prepared for the hypocotyl length assay. In the regulation of high light × high temperature and high light × low temperature, the hypocotyl length in Col‐0 and *phyB‐9* decreased significantly as water potential decreased, but the *phyB‐9* seedlings were more sensitive to low water potential than Col‐0 (Figs S2 and S3), while hyposensitivity to water potential was observed in *phyB‐9* under the regulation of low light × high temperature and low light × low temperature (Figs S4 and S5). The results indicated that PhyB could play key roles in regulating hypocotyl growth by integrating environmental signals. The anova comparison revealed that the influence of water potential under the background of high light × high temperature, high light × low temperature, low light × high temperature, and low light × low temperature reached significant level, respectively (Table S8‐S11).

## Discussion

### Hypocotyl elongation under the combined effect of multi‐environmental factors

To adapt to the surrounding fluctuations, higher plants exhibited great plasticity in architecture (Patel and Franklin 2014, Quint et al. 2016, Legris et al. 2017, Casal and Questa 2018). As a vital energy source and information signal, light regulates plant growth and development throughout life (Hersch et al. 2014 **,** Xu et al. 2018). Seedlings of *B. rapa* always strongly elongate to compete for better light harvest (Procko et al. 2014). Temperature is another major environmental factor that dominates distribution and seasonal behaviors of the seedlings. Hypocotyl elongation induced by high temperature was conserved among crop species, such as *A. thaliana*, *Helianthus annuus*, *B. oleracea* and *S. lycopersicum* (Kurepin et al. 2010, Quint et al. 2016). Low water potential caused by water deficit and high salinity restricted plant growth and production (Munns 2002, Chen et al. 2005). For dark‐grown plants, hypocotyl elongation was significantly restricted by low water potential, and the elongation was ceased when the water potential decreased to −1 MPa in *A. thaliana* (Cm et al. 2000 **,** Wu et al. 2000). In addition, environmental factors not only regulate hypocotyl growth in isolation but also in coaction (de Wit et al. 2014, Toledo‐Ortiz et al. 2014, Lorenzo et al. 2016). As reported, the classic light response suggested that the suppression of hypocotyl growth was strictly temperature‐dependent, and a reverse response from inhibition to promotion was induced by the shift in temperature (Johansson et al. 2014). In our research, low light, high temperature and high water potential regulated hypocotyl growth in both isolation and coaction (Fig. 2). As the light intensity decreased, the hypocotyl length increased incrementally and significantly, and the maximum increase occurred under the combined effects of high temperature and water potential, and vice versa, which was similar to the findings of a previous report (Kurepin et al. 2010).

### Proteins identified in the elongating hypocotyl

The proteomic study of hypocotyl elongation at different developmental stages using gel‐dependence has been studied (Irshad et al. 2008, Jamet et al. 2009). However, the metabolism that occurs in other cell parts was still unclear, because most published research primarily focuses on cell wall proteins. In addition, the protein number was strictly limited by technology. For example, Irshad et al. (2008) used 2‐dimensional analysis to identify 137 proteins in cell wall protein dynamics in elongating cells. Since then, the application of high‐throughput LC/MS–MS improved the protein numbers in proteomics research (Duruflé et al. 2017, Lin et al. 2017). The proteomic research on hypocotyl elongation resulted in the detection of 1209 proteins in *A. thaliana* (Novak et al. 2015). In our study, LC/LC–MS/MS technology was adopted, and approximately 7200 proteins were identified in elongating hypocotyls. This information enabled us to analyze the mechanism of hypocotyl elongation in response to light intensity, temperature and water potential.

### Underlying mechanism of hypocotyl elongation

To explore proteins and pathways responding to multifactors regulating hypocotyl elongation, the proteomic profiles were detected using a high‐throughput iTRAQ approach. A total of 378 proteins were differentially expressed under the combined effects of light intensity, temperature and water potential (Fig. 5). The synergistically regulatory mechanism included the process of signal transduction, cell wall biosynthesis, cytoskeleton organization, carbohydrate metabolism and cell wall organization.

### Signal transduction

The comparative proteomic profiles revealed that nine DEPs responsive to the three environmental factors were associated with signal transduction (Fig. S6). Three proteins [PhyB‐, Early Flowering 4‐ (ELF4‐), Protein Phosphatase Type‐2C‐ (PP2C‐)] had lower abundance, which had been reported to be key factors in the regulation of hypocotyl elongation mediated by shade avoidance and high temperature (Hersch et al. 2014, Lu et al. 2015 **,** Xu et al. 2018). The other six proteins dispersed in the downstream showed higher abundance, and some were reported to be closely related to cell wall extensity during hypocotyl elongation, such as Expansin A11 (Irshad et al. 2008). These results indicated that low light, high temperature and high water potential facilitated hypocotyl elongation in *B. rapa* by improving the extensity of cell wall and water‐uptake.

### Cytoskeleton organization

The plant cytoskeleton plays a key role in a number of cellular processes, such as cell morphogenesis, organogenesis and development (Chaudhry et al. 2007, Deeks et al. 2007). The arrays of microfilaments and microtubules govern the process of cell division, elongation, cell wall deposition and cellulose direction in the inner wall (Barrero 2002). The comparative proteomic profiles between low light vs high light, high temperature vs low temperature, and high water potential vs low water potential revealed that the quantities of 10 proteins fluctuated (Fig. S7). The actin monomers (ACTIN 2, ACT2), tubulin monomers TUBULIN beta‐9 chain, (Tublin 9, TUB 9) and their associated proteins Lin‐Inl‐Mec (LIM) domain‐containing protein 1 and PCAP1 were all upregulated by the three environmental factors, which were reported to regulate cell elongation through facilitating filament treadmilling (Sedbrook et al. 2004 **,** Harries et al. 2005 **,** Li et al. 2011). These findings indicated that the pathway of hypocotyl cell elongation via cytoskeleton organization was conserved among regulation by the three environmental factors.

### Carbohydrate metabolism and cell wall organization

The protein expression files in the hypocotyls treated with the three environmental factors had a higher abundance of six proteins and a lower abundance of five proteins associated with carbohydrate metabolism and cell wall organization (Fig. S8). Only two of these proteins were involved in sucrose and starch degradation, which provide carbon skeletons for cell wall organization. One key enzyme responsible for cellulose synthesis from GUANOSINE DIPHOSPHATE (GDP)‐glucose was upregulated by low light, high temperature and high water potential, while another protein involved in cellulose degradation was downregulated. The degree of pectin methyl‐esterification restricted cell growth, and the low level of methyl‐esterification led to reduced cell elongation (Micheli 2001, Derbyshire et al. 2007). The pectin methylesterase inhibitor, functioning in regulating the methyl‐esterification level, was differentially expressed. Simultaneously, a pectin‐degrading enzyme was also differentially expressed under the influence of light intensity, temperature and water potential, and the role of this enzyme in remodeling cell wall during cell elongation had been demonstrated (Domingo et al. 1998, Marín‐Rodríguez and Seymour 2002). Lignin, as a phenolic compound, consists of three basic subunits: p‐hydroxyphenyl (H), guaiacyl (G) and syringyl (S) monolignols (Bonawitz and Chapple 2010). As an important component of cell walls, lignin primarily regulated cell growth by reducing cell wall extensity and affecting auxin transport (Fan et al. 2006, Besseau et al. 2007). In this study, five enzymes involved in monolignol synthesis were detected, and the first key rate‐limiting enzyme, PHENYLALANINE AMMONIA‐LYASE 1, was negatively related to hypocotyl elongation, while the other three enzymes, 4‐COUMARATE‐CoA LIGASE 1, CAFFEOYL‐O‐METHYLTRANSFERASE and PEROXIDASE, were upregulated during the growth deficiency caused by the shortage of lignin. Another protein, β‐GLU, involved in the catabolism of cinnamate was negatively associated with hypocotyl length, which indicated the shortage of metabolic intermediates for lignin synthesis. In conclusion, all three environment factors, low light, high temperature and high water potential, facilitated hypocotyl growth by promoting cellulose synthesis and pectin degradation, and maintaining high level of pectin methyl‐esterification and the balance of lignin metabolism.

### PhyB: emerging hubs in light intensity, temperature and water potential signaling in the regulation of hypocotyl elongation

PhyB, known as a photoreceptor, had been characterized elaborately with its function in photomorphogenesis, including repressing hypocotyl cell elongation by repressing hormone biosynthesis and signaling (Halliday and Davis 2016, Pearce et al. 2016 **,** Xu et al. 2018). PhyB synthesized in an inactive form (Pr), which turned into an active form and transferred into the nucleus from the cytoplasm after excitation (Medzihradszky et al. 2013, Klose et al. 2015, Park et al. 2017). In the nucleus, PhyB associated with its downstream signaling components (PIF4, COP1 and HY5) regulates hypocotyl elongation (Lu et al. 2015, Choi and Oh 2016, Martin et al. 2016, Ezer et al. 2017). In response to shade and high temperature, PhyB was deactivated and translocated to the cytoplasm, inducing the accumulation of PIF4/5/7 and activating the expression of auxin biosynthetic‐related genes to promote indoleacetic acid (IAA) production and hypocotyl cell elongation (Sun et al. 2012 **,** Ren and Gray 2015). And the biosynthetic genes of gibberellin were downregulated by the over‐expression of *PhyB* in *A. thaliana*, which promoted hypocotyl cell elongation primarily by inducing the degradation of the DELLA protein and the accumulation of PIF4/5/7 (Song et al. 2015). In addition to regulate hormones biosynthesis, PhyB also participated in hormone signaling, which interacted directly with the auxin/indoleacetic acid (Aux/IAA) proteins to inhibit auxin signaling transduction in regulating hypocotyl cell elongation (Xu et al. 2018). In another pathway, PhyB can attenuate the influence of brassinolide (BR) on promoting hypocotyl elongation through decrease in the accumulation of PIF4, which interacts with the downstream BR responsive factors, such as *BZR1* and *BES1* (Bai et al. 2012). In previous study, the functions of PhyB were conserved among *B. rapa*, *A. thaliana*, tobacco (*Nicotiana tabacum* L.) and soybean (*Glycine max*) (Wagner et al. 1991, Halliday et al. 1997, Wu et al. 2011, Song et al. 2015). In addition, a high homology of sequence and structure between BrPhyB and AtPhyB was discovered (Song et al. 2015), so we studied the function of BrPhyB by analyzing the changes in hypocotyl length of *phyB‐9* and Col‐0 in *A. thaliana*.

Plants have developed elaborate systems to integrate environment cues provided by light intensity, temperature and water potential to adjust their growth pattern (Kristie and Jolliffe 1987, Casal 2013, Toledo‐Ortiz et al. 2014). PhyB was identified as an emerging hub in light and temperature signaling in the current study (Lorenzo et al. 2016, Casal and Questa 2018). In addition, recent research identified that PhyB is also involved in regulating drought tolerance in *A. thaliana* by reducing abscisic acid (ABA) content and enhancing the sensitivity to ABA, which suppressed hypocotyl elongation primarily by regulating the activity of H^+^‐ATPase (Hayashi et al. 2014, Cohen et al. 2015). In our research, the protein abundance of PhyB changed significantly in response to light intensity, temperature and water potential (Table S6), and the mutant seedlings of *phyB‐9* exhibited hypersensitivity and hyposensitivity to water potential perturbations under high light and under low light conditions, respectively, indicating that PhyB may play a multifunctional role in regulating hypocotyl growth both on ABA sensitivity and contents. All the results above indicated that PhyB may be the hub of environmental factors in regulating hypocotyl elongation.

## Author contributions

Q.S. and H.W. designed the experiment. H.W. did the experiment and analyzed the data. H.W. wrote the manuscript and Q.S. reviewed it.

## Supporting information


**Fig. S1.** Molecular weight distribution of the total proteins.
**Fig. S2.** Hypocotyl phenotypes of *A. thaliana* seedlings in response to water potential under the combined effects of high light and high temperature.
**Fig. S3.** Hypocotyl elongation in response to water potential under the combined effects of high light and low temperature.
**Fig. S4.** High water potential promotes hypocotyl elongation dependent on PhyB under low light and high temperature.
**Fig. S5.** Hypocotyl elongation induced by high water potetnial depends on PhyB under low light and low temperature.
**Fig. S6.** Overview of DEPs associated with signals transduction.
**Fig. S7.** Overview of DEPs related to cytoskeleton organization.
**Fig. S8.** Overview of DEPs associated with cell wall construction.

**Table S1.** Physicochemical properties assay of PEG‐6000 solutions.
**Table S2.** Primer sequences used for qRT‐PCR.
**Table S3.** Interaction analysis among light intensity, temperature and water potential.
**Table S4.** Basic protein identification information.
**Table S7.** Interaction analysis among environmental factors and genotype.
**Table S8.** Interaction analysis under the combined effects of high light and high temperature.
**Table S9.** Interaction analysis between under the combined effects of high light and low temperature.
**Table S10.** Interaction analysis under the combined effects of low light and high temperature.
**Table S11.** Interaction analysis under the combined effects of low light and low temperature.

**Table S5.** Information of 7259 proteins.

**Table S6.** The 378 cross‐responsive DEPs and their functional classification.
