# Supplementary material for: Identification and functional analysis of proteins in response to light intensity, temperature and water potential in Brassica rapa hypocotyl
Source: Physiol Plant. 2019 Jan 10;167(1):48–63. doi: 10.1111/ppl.12865 (PMC6850590; doi:10.1111/ppl.12865)
Supplement: Supplementary file 1 — Fig. S1. Molecular weight distribution of the total proteins. Fig. S2. Hypocotyl phenotypes of A. thaliana seedlings in response to water potential under the combined effects of high light and high temperature. Fig. S3. Hypocotyl elongation in response to water potential under the combined effects of high light and low temperature. Fig. S4. High water potential promotes hypocotyl elongation dependent on PhyB under low light and high temperature. Fig. S5. Hypocotyl elongation induced by high water potetnial depends on PhyB under low light and low temperature. Fig. S6. Overview of DEPs associated with signals transduction. Fig. S7. Overview of DEPs related to cytoskeleton organization. Fig. S8. Overview of DEPs associated with cell wall construction. [file PPL-167-48-s001.docx]

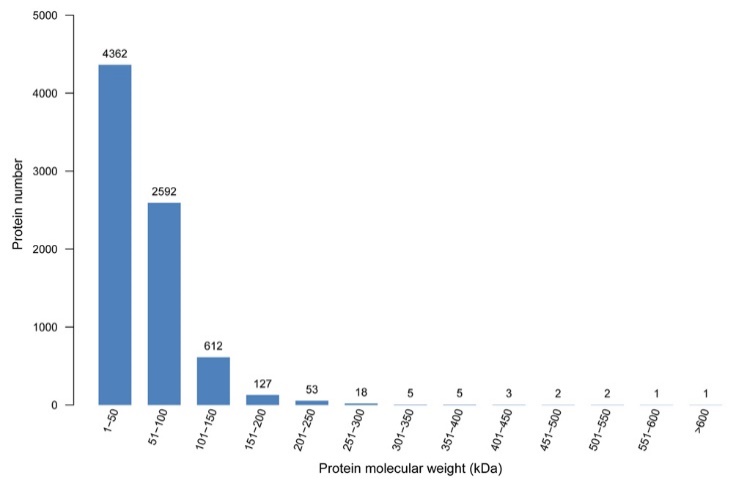


**Fig. S1.** Molecular weight distribution of the total proteins identified in hypocotyls of *B. rapa*.


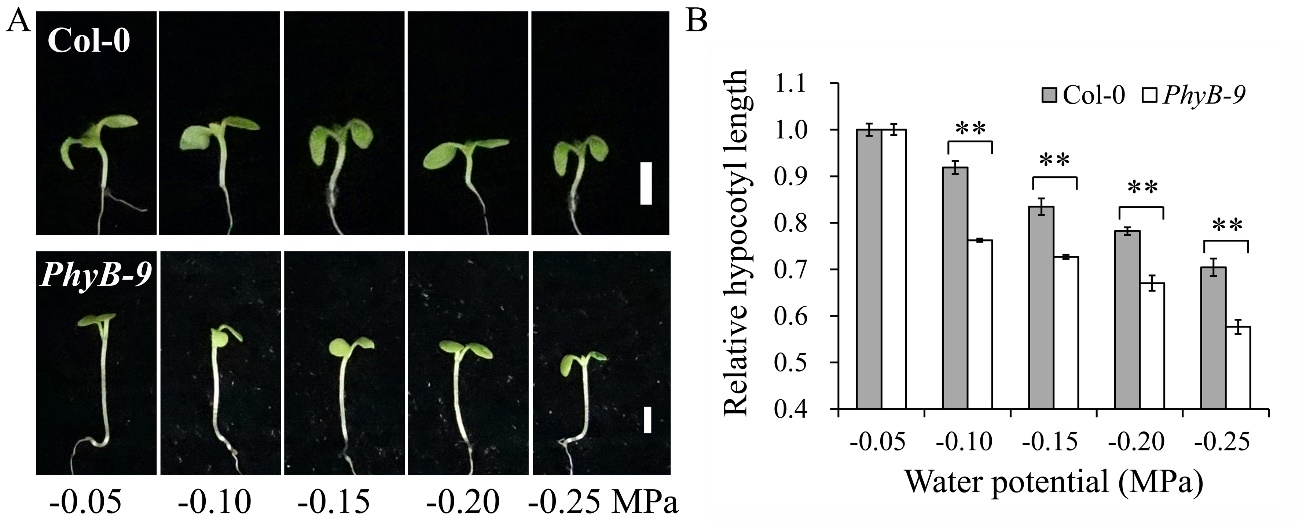


**Fig. S2.** Hypocotyl phenotypes of *A. thaliana* seedlings in response to water potential under the combined effects of high light and high temperature. (A) Seedling phenotypes in Col-0 and *phyB-9* in response to water potential. (B) The increase in hypocotyl length induced by high water potential in Col-0 and *phyB-9*. Light intensity was 250 μmol·m^-2^·s^-1^ (16-h photoperiod); temperature was 29°C. Scale bars in picture (A) represent 2.5 mm. Error bars in picture (B) represent SE. Hypocotyl lengths of seedlings were measured and taken photographs at 120 h after water potential treatment.


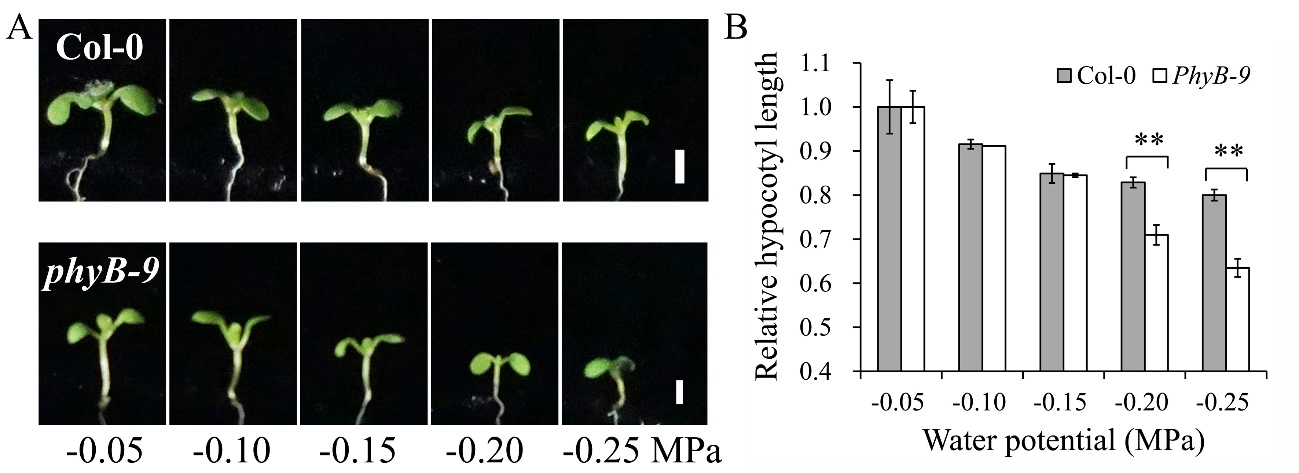


**Fig. S3.** Hypocotyl elongation in response to water potential under the combined effects of high light and low temperature. (A) Seedling phenotypes of Col-0 and *phyB-9* in response to water potential under the combined effects of high light and low temperature. (B) Statistical data of hypocotyl length in Col-0 and *phyB-9* by water potential treatment. Light intensity was 250 μmol m^-2^ s^-1^ (16-h photoperiod); temperature was 21°C. Scale bars in picture (A) represent 2.5 mm. Error bars in picture (B) represent SE. Hypocotyl lengths of seedlings were measured and taken photographs at 120 h after water potential treatment.


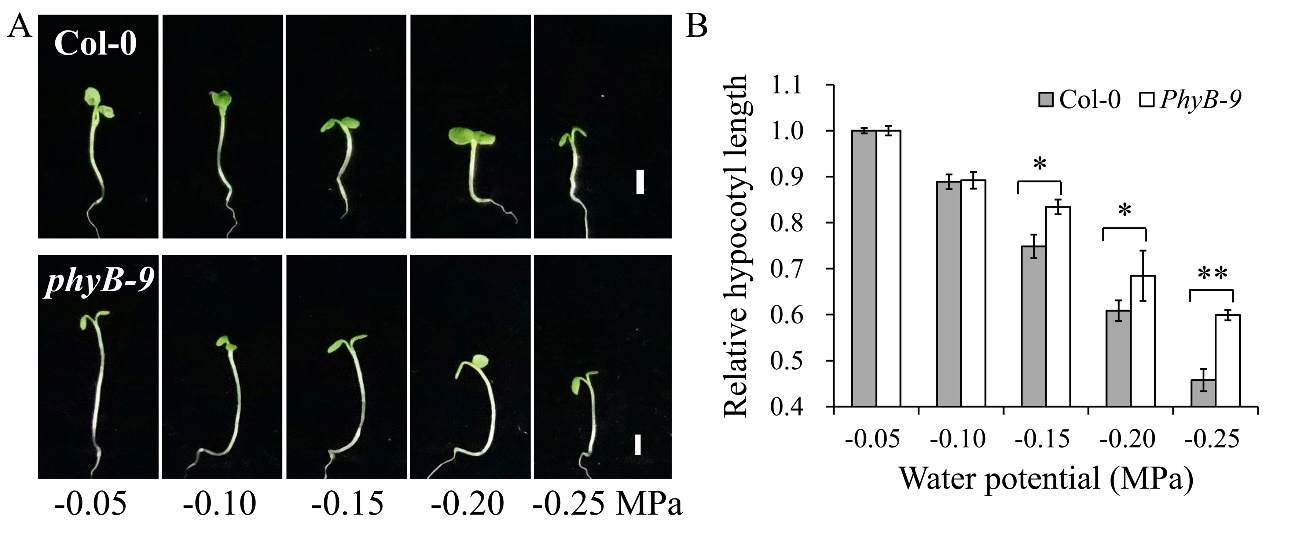


**Fig. S4.** High water potential promotes hypocotyl elongation dependent on phyB under low light and high temperature. (A) Seedling phenotypes in Col-0 and *phyB-9* with different water potential treatment. (B) Statistical data of hypocotyl length in Col-0 and *phyB-9* by water potential treatment under the influence of low light and high temperature. Light intensity was 50 μmol m^-2^ s^-1^ (16-h photoperiod); temperature was 29°C. Scale bar = 5 mm. Error bars in picture (B) represent SE. Hypocotyl lengths of seedlings were measured and taken photographs at 120 h after water potential treatment.


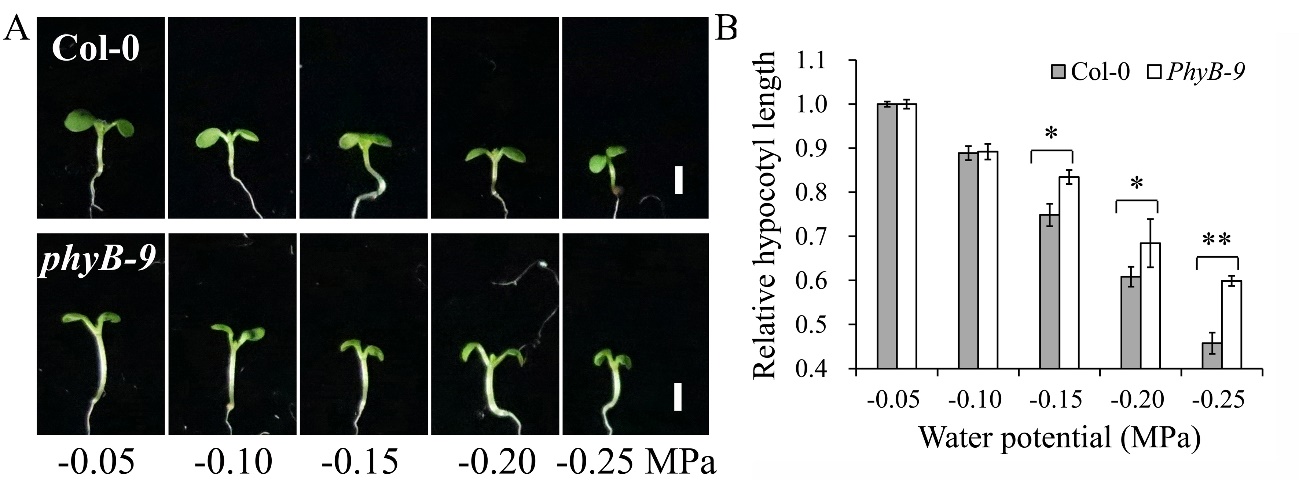


**Fig. S5.** Hypocotyl elongation induced by high water potetnial depends on phyB under low light and low temperature. (A) Photograph of Col-0 and *phyB-9* seedlings with different water potential treatments. (B) Statistical data of hypocotyl length in Col-0 by water potential treatment. Light intensity was 50 μmol m^-2^ s^-1^ (16-h photoperiod); temperature was 21°C. Scale bar = 5 mm. Error bars in picture (B) represent SE. Hypocotyl lengths of seedlings were measured and taken photographs at 120 h after water potential treatment.


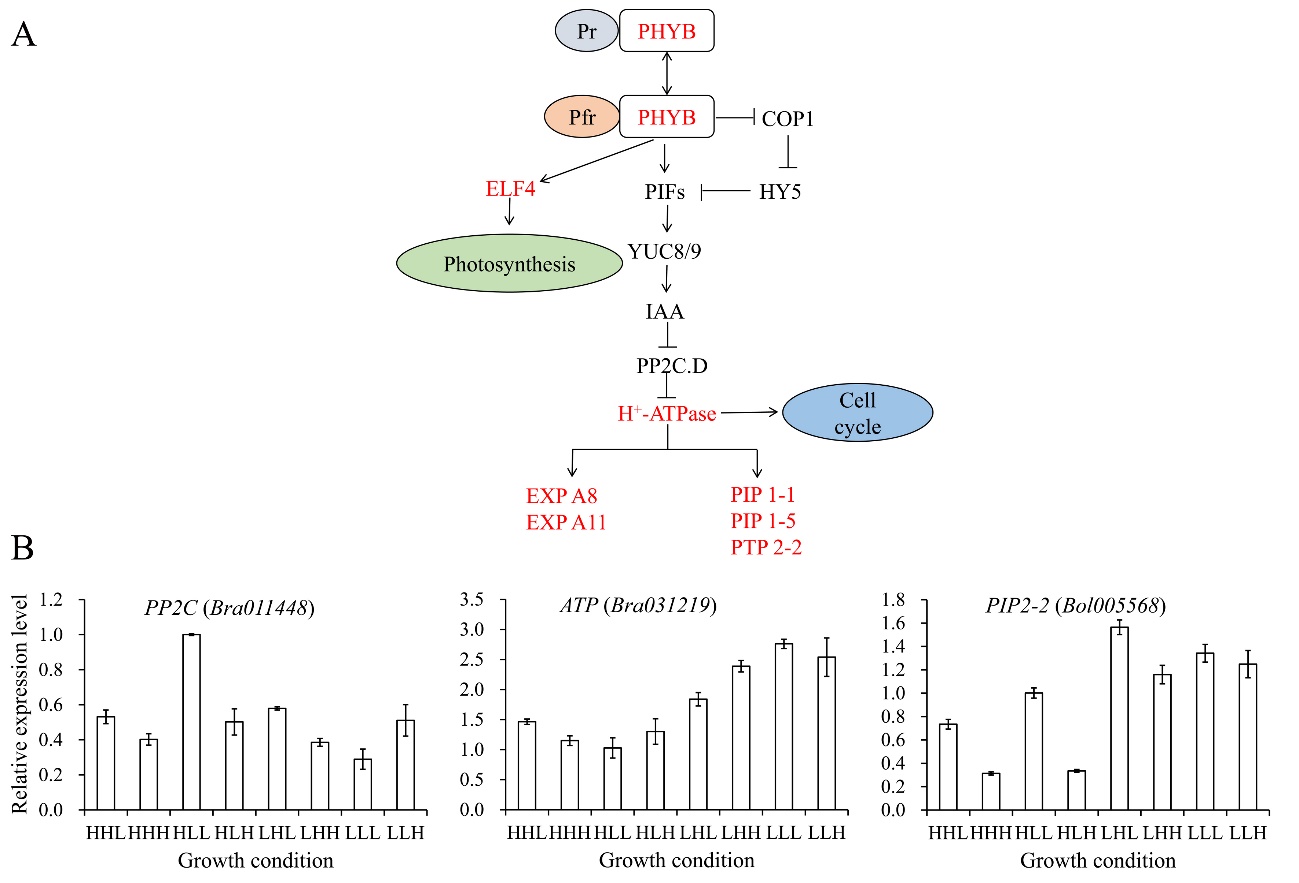


**Fig. S6.** Overview of DEPs associated with signals transduction. (A) Siganls transduced by phyB are illustrated. Responsive DEPs are marked in red. (B) Relative expression level of DEPs at mRNA level. The data in B represents the means of three replicates ± SD.


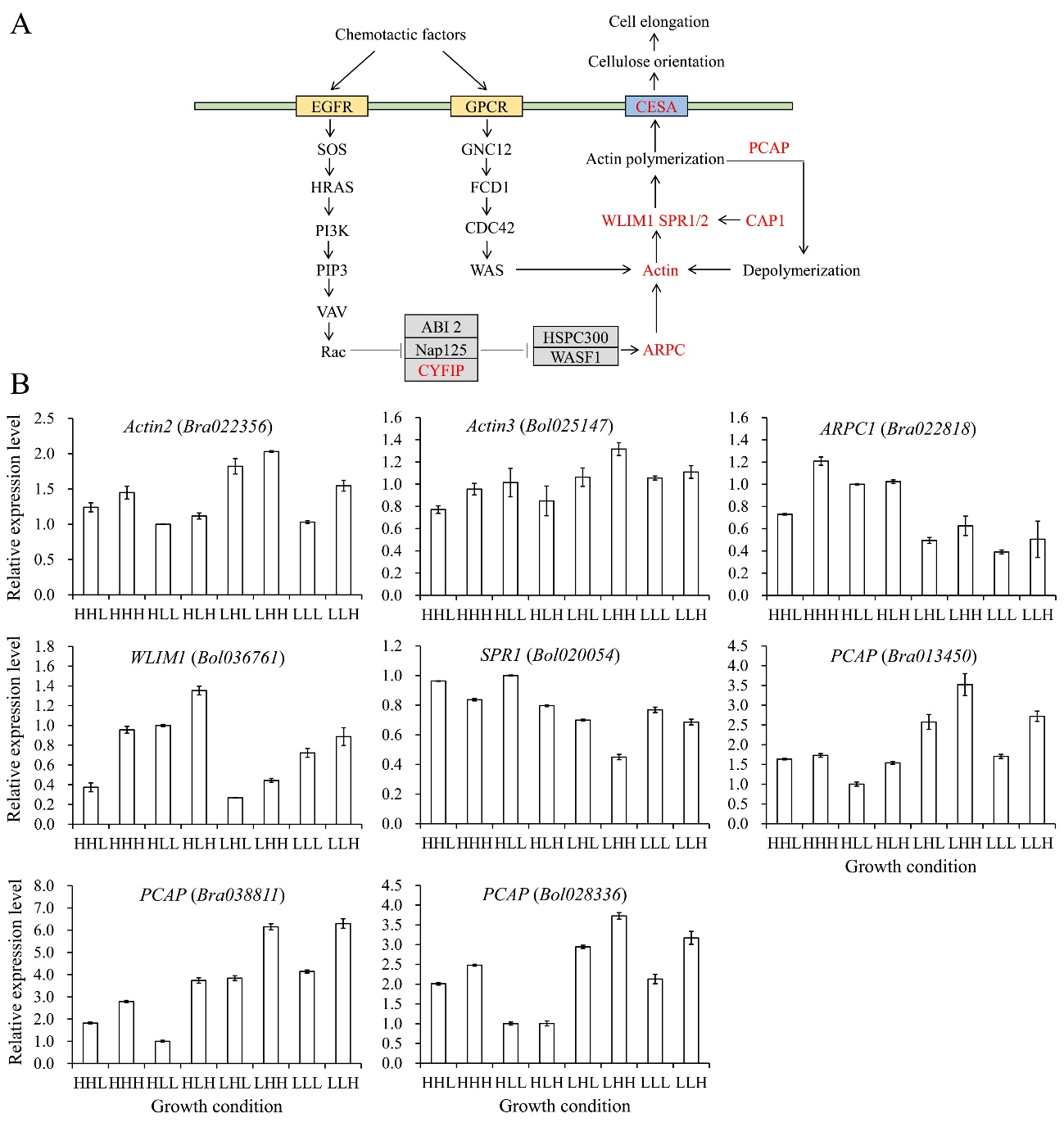


**Fig. S7.** Overview of DEPS related to cytoskeleton organization. (A) Process of cytoskeleton organization. The DEPs are marked in red. (B) Relative expression of DEPs in cytoskeleton organization at mRNA level. Error bars in picture (B) represent SE.


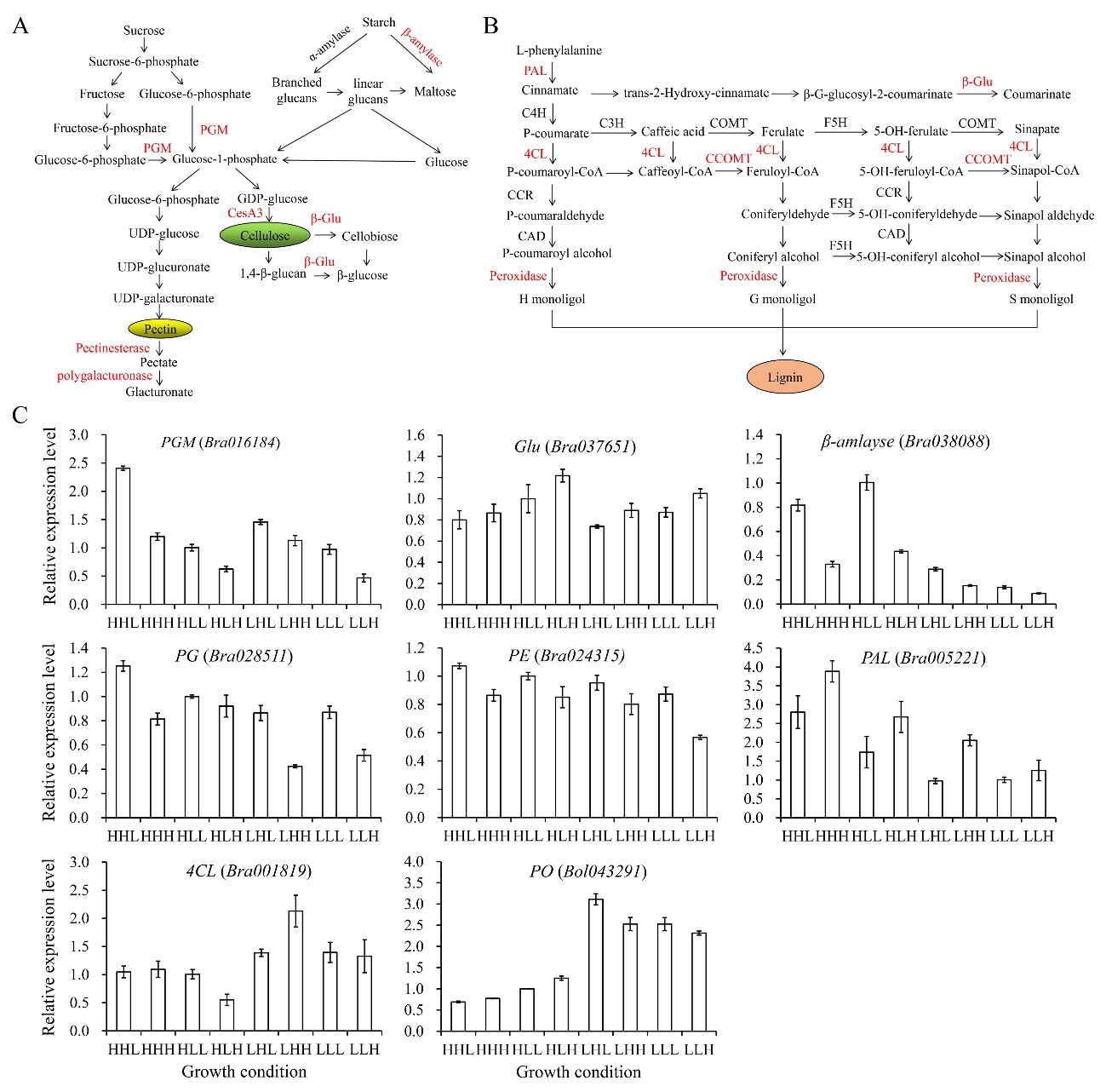


**Fig. S8.** Overview of DEPs associated with cell wall construction (A) Pathways in cell wall construction in response to light, temperature and water potential. DEPs responsive to the three environmental factors are marked in red (B) Relative expression level of DEPs in cell wall construction at mRNA level. The data in B represents the means of three replicates ± SD.
