# Supplementary material for: Identification and functional analysis of proteins in response to light intensity, temperature and water potential in Brassica rapa hypocotyl
Source: Physiol Plant. 2019 Jan 10;167(1):48–63. doi: 10.1111/ppl.12865 (PMC6850590; doi:10.1111/ppl.12865)
Supplement: Supplementary file 2 — Table S1. Physicochemical properties assay of PEG‐6000 solutions. Table S2. Primer sequences used for qRT‐PCR. Table S3. Interaction analysis among light intensity, temperature and water potential. Table S4. Basic protein identification information. Table S7. Interaction analysis among environmental factors and genotype. Table S8. Interaction analysis under the combined effects of high light and high temperature. Table S9. Interaction analysis between under the combined effects of high light and low temperature. Table S10. Interaction analysis under the combined effects of low light and high temperature. Table S11. Interaction analysis under the combined effects of low light and low temperature. [file PPL-167-48-s002.docx]

**Supplementary Material**

**Supplementary Table**

**Table S1. Physicochemical property of irrigating solutions**

| PEG concentration (%) | Osmotic potential (Mpa) | Water potential (Mpa) |
| --- | --- | --- |
| 0 | 0.00 | -0.05 |
| 4 | -0.36 | -0.09 |
| 8 | -1.03 | -0.15 |
| 12 | -2.01 | -0.27 |
| 16 | -3.29 | -0.38 |

Note: The osmotic potential of solutions was calculated according to Michel and Kaufmann (1973) in case that temperature was 25°C.

Reference

Michel BE, Kaufmann MR. (1973) The Water potential of Polyethylene Glycol 6000. Plant physiology 51 (5):914.

**Table S2. Primer sequences used for RT-qPCR**

| Protein name | Accession No. | Forward primers sequence (5'-3') | Reverse primers, sequence (5'-3') |
| --- | --- | --- | --- |
| 13-3-3 | Bra040592 | CAATCTCGCCAAACAGG | CAGAAGTCCAGAGGGTCA |
| 4CL | Bol031583 | CCGAATCTTTACTTCCACA | AGCAACCGTCACTTTACAC |
| 4CL | Bra001819 | ACAGATCCAGAGCCTTCTCA | ATCCCTAGCCGATGTATCCC |
| Actin2 | Bra022356 | GGGATGAACCAGAAGGATG | TCAGGAGCAATACGGAGC |
| Actin3 | Bol025147 | GGAATGGTCAAGGCTGGTT | TGCCGTGCTCAATAGGGT |
| ARPC1 | Bra022818 | GGTGATAGGCGTTGGTTA | GCTTCGTTCGCTGTTGTA |
| ATP | Bol001514 | TGAAGCAAGCCAAGGAAG | GCACCCGAGTCTCCACTT |
| ATP | Bra012809 | TGTCCAGGAGATAAGCAA | CCAGTAGTCGTTACGGATT |
| ATP | Bra031219 | ATGCTCGTGCTTACCTCT | CCTTTCTTGTCGCTTCTC |
| ATP11 | Bol027311 | ATCGCTTGGTTCAGTCAG | TCGGGAGATTTAGTCTTGG |
| CAP | Bol034684 | TGAAAGCTGACGCTATGACT | AGAACTCAGCCATCTGCCAA |
| CCOMT | Bol006844 | CATATCCAAGAGAGCATGAGGAG | CACCGAGTTCTAGCGTATCTT |
| CesA3 | Bra028768 | TCTGTGGATTCCCTGTTTG | CTCCTTCTGCGGGTAGTT |
| ELF4 | Bol016797 | TCACCAGTCAAGAATGGCAGAT | TGAAACGCCGAGGAGAAA |
| ELF4 | Bra000165 | GAGGAACGTGGCAGAGG | TGTTTCCGTTGAGTTCCTGG |
| EXPA8 | Bol006881 | GAAGAAAGGAGGGATAAGG | CTGGTCGTTGAGGTAAGAAT |
| EXPA11 | Bol023607 | CTAACTGGCAATCAAACG | ACTGAACCCTGGAAGAAT |
| FER | Bol007379 | TCAGCCAACACTCAATCCC | CAACCTCCTGCTCACCTTCT |
| FYPP1 | Bra014246-2 | TTTATGGGAGATTTCGTTG | CTTCCTTTGGCATTCGT |

| Glucodase | Bol033912 | GCGTATCAGGTTGAAGGT | ATCGCCGTTATCGTTGTT |
| --- | --- | --- | --- |
| Glucodase | Bra018969 | CTGGTGGTAAACTGCCTGTG | CCGAACCTCGCCTTGTAT |
| Glucodase | Bra037651 | CGGCGGAAGATAGGTTAG | TTCGCAGTGAAAGTAGGC |
| H2A.1 | Bol005821 | GTCGTATCGCTCGTTTCCTG | CCTCGTCTTCTTGTTATCCCTC |
| H2A.3 | Bol039718 | CCCGTTGGTCGTATCGC | CCTCGTCGTTCCTCACAGC |
| H2B.10 | Bra002436 | AGAAGAGCGTGGAGACCTAC | CCTCGAAGTGATCGTGGG |
| H2B.11 | Bol036345 | ACCCAAGACTAGCGGCA | CTTGTTGTAGTGGGCGAGAC |
| H3.3 | Bol028862 | GCTCGTAAGTCTACAGGAGG | GCCTGTAACGATGTGGCTTC |
| LHCA2 | Bol037491 | CCCAATCTTCCCAAACAAC | GAACCAAGCACCCATCACT |
| LHCA4 | Bra018144 | AACTTCGCTCCTACGCT | GTCCCTTTCCAGTCACATT |
| LHCB1 | Bra022976 | AAGAACGGAAGGTTGGCTATG | AGGGACGAAGTTGGTGGC |
| LHCB4 | Bra029732 | GAGAAGCGTTTGTATCCAGG | GTGAGTCGCCCAGTTGTT |
| LHCB5 | Bol007720 | GCCTTTGAACTTATCCACGC | TCAGCAACTACAGCGAGAAC |
| MCM3 | Bol006344 | GCGAGATTTCATCCAGTTCCT | AGATTCGCGGAAGTGGGTAT |
| PAL | Bra005221 | AGCCTGTGGTGAATCTCGG | CCATAACTATCAGTGCCTTTGC |
| PATL1 | Bra003892 | ACACCGTCCAATGGCGTAA | GGAGCGTTCCTGAAGTCG |
| PATL2 | Bra040854 | GGCCGTAGTTAAATCCTCAG | TGCCTCGTTGGTTGGTTC |
| PCAP | Bol028336 | GAGGCTCTTGTCGGCATAG | CAGGTACTTCCCGTGTCTTAA |
| PCAP | Bra013450 | GAGGCTCTTGTCGGCATAG | CAGGTACTTCCCGTGTCTTAA |
| PCAP | Bra038811 | GAGGAGCACAAACCAGAGG | TCTTCGTCTCTGGTGCCTT |
| PE | Bra002328 | AAATCCGACCCAAGAAGC | TGAACCGTTAGCCGAAGA |
| PE | Bra024315 | GCAACCGCCACTTACATAG | TGAACCGTTAGCCGAAGA |
| PG | Bra028511 | GCGGAATCCAGAATGTC | TTGATCGTTATGCCTCGT |
| PGM | Bra016184 | ACCCTGAACACTACGACACT | AAGATAGAGCGAACCGAAT |
| PHYB | Bra022192 | ATTATCCAGCTACTGATATTCC | GACCCAACCAAGCACATA |
| PIP1-1 | Bra014437 | CCAAGATGGAAGGCAAGG | CGGTGGTGGCTCGTTAT |
| PIP1-5 | Bol042093-12 | GCTTTAACAGTCTTGGGGGT | CACAGATAGCTCCAAGGCAC |
| PIP2-2 | Bol005568 | CGACACTAAGGCAGGAGG | GCCAAGAACAAGCCGAAC |
| PO | Bol043921 | GACGTGCCCTAACGCATCT | GCTTCCACTGTCGTCAAGCA |
| PO | Bra023099 | GATGCGTCACTGCTACTA | GTGCGTCTTTGATAACCT |
| PP2C | Bra011448 | TCTGATGGGCTCTTTGAC | CACCTCTTGTGCCTTTGC |
| Rubisco | Bol024765 | ATGGCTTCCTCTATGCTC | CTTCTTCCCGACTGGTG |
| Rubisco | Bra025431 | GAGTTGGAGCACGGATTTG | TGGACCTGACGGTTGTTGT |
| Rubsico | Bol038967 | AGTAACTTCTCAACCCGTAG | GTGGTAGCATCGTCCTTT |
| SNEAR | Bol010435 | AAGAAACGAGGTGTCAGG | AAACTTGGGCTTGGGTC |
| SPR1 | Bol020054 | ATGGGTCGTGGAAACAGC | CGAAGGACGGTCAGTGATG |
| SPR2 | Bra026382 | TCTCTTCTCTCACCCGCATT | GCGAGTTTGGAGATTGAGGT |
| WLIM1 | Bol036761 | AAAACCGTGTACCTCGTC | AATCTTTGGTGTCCCTTC |
| β-amlayse | Bra038088-2 | GCACGCTGACCAAGATTAC | GTATCCCACTTGAATGCCCC |
| β-amylase | Bra038088 | GCACGCTGACCAAGATTAC | TCCTTCTCTTATTTTGTTTGACGA |

**Table S3. Interaction analysis among light intensity, temperature and water potential**

| Factor | Mean square | P value |
| --- | --- | --- |
| Block | 0.0025 | 0.5216 |
| Light intensity | 1.0458 | ＜0.001 |
| Temperature | 0.2185 | ＜0.001 |
| Water potential | 0.3725 | ＜0.001 |
| Light intensity × Temperature | 0.026 | 0.0188 |
| Light intensity × Water potential | 0.0012 | 0.5766 |
| Temperature × Water potential | 0.1764 | 0.0463 |
| Light intensity × Temperature × water potential | 0.0287 | 0.0144 |

**Table S4.** **Basic protein identification information in the three replications**

| **Replication No.** | **Total Spectrum** | **Identified Spectrum** | **Peptide number** | **Protein group number** |
| --- | --- | --- | --- | --- |
| 1 | 399450 | 128802 | 40982 | 7783 |
| 2 | 352363 | 100299 | 41358 | 7387 |
| 3 | 362523 | 112764 | 44799 | 7984 |
| Average | 371445 | 113955 | 42380 | 7718 |

**Table S7. Interaction analysis among light intensity, temperature, water potential and genotype on regulation hypocotyl elongation**

| Factor | Mean square | P value |
| --- | --- | --- |
| Water potential | 8.383 | 2.684E-22 |
| Temperature | 32.637 | 1.749E-31 |
| Light intensity | 45.552 | 8.953E-34 |
| Genotype | 43.017 | 2.219E-33 |
| Water potential × Genotype | 0.230 | 3.012E-04 |
| Temperature × Genotype | 0.599 | 2.266E-07 |
| Light intensity × Genotype | 0.594 | 2.430E-07 |
| Water potential × Temperature | 1.993 | 2.496E-13 |
| Water potential × Light intensity | 0.691 | 5.672E-08 |
| Temperature × Light intenisity | 2.765 | 2.993E-15 |
| Water potential × Temperature × Genotype | 0.065 | 0.039 |
| Water potential × Light intensity × Genotype | 0.156 | 0.002 |
| Temperature × Light intenisity × Genotype | 2.009 | 2.242E-13 |
| Water potential × Temperature × Temperture | 0.919 | 2.939E-09 |
| Water potential × Temperature × Temperture × Genotype | 0.050 | 0.122 |

**Table S8. Interaction analysis between water potential and genotype on regulation hypocotyl elongation under the combined effects of high light and high temperature**

| Factor | Mean square | P value |
| --- | --- | --- |
| Water potential | 4.157 | 2.442E-18 |
| Genotype | 99.408 | 7.620E-28 |
| Water potential × Genotype | 1.464 | 6.366E-14 |

**Table S9. Interaction analysis between water potential and genotype on regulation hypocotyl elongation under the combined effects of high light and low temperature**

| Factor | Mean square | P value |
| --- | --- | --- |
| Water potential | 0.624 | 3.349E-11 |
| Genotype | 1.850 | 9.395E-12 |
| Water potential × Genotype | 0.130 | 1.648E-05 |

**Table S10. Interaction analysis between water potential and genotype on regulation hypocotyl elongation under the combined effects of low light and high temperature**

| Factor | Mean square | P value |
| --- | --- | --- |
| Water potential | 16.973 | 1.114E-14 |
| Genotype | 67.230 | 2.050E-16 |
| Water potential × Genotype | 0.076 | 0.612 |

**Table S11. Interaction analysis between water potential and genotype on regulation hypocotyl elongation under the combined effects of low light and low temperature**

| Factor | Mean square | P value |
| --- | --- | --- |
| Water potential | 2.053 | 1.122E-08 |
| Genotype | 9.850 | 4.307E-11 |
| Water potential × Genotype | 0.208 | 0.027 |
